# Specific binding of *Bacillus thuringiensis* Cry1Ea toxin, and Cry1Ac and Cry1Fa competition analyses in *Anticarsia gemmatalis* and *Chrysodeixis includens*

**DOI:** 10.1038/s41598-019-54850-3

**Published:** 2019-12-03

**Authors:** Yolanda Bel, Marc Zack, Ken Narva, Baltasar Escriche

**Affiliations:** 1 ERI de Biotecnología y Biomedicina (BIOTECMED), Department of Genetics, , Universitat de València, 46100 Burjassot, Spain; 2 Dow AgroSciences, Indianapolis, Indiana, USA

**Keywords:** Environmental biotechnology, Applied microbiology

## Abstract

*Anticarsia gemmatalis* (velvetbean caterpillar) and *Chrysodeixis includens* (soybean looper) are two important defoliation pests of soybeans. In the present study, we have investigated the susceptibility and brush border membrane-binding properties of both species to *Bacillus thuringiensis* Cry1Ea toxin. Bioassays performed in first-instar larvae demonstrated potent activity against both soybean pests in terms of mortality or practical mortality. Competition-binding studies carried out with ^125^Iodine-labelled Cry1Ea, demonstrated the presence of specific binding sites on the midgut brush border membrane vesicles (BBMV) of both insect species. Heterologous competition-binding experiments indicated that Cry1Ea does not share binding sites with Cry1Ac or Cry1Fa in either soybean pest. This study contributes to the knowledge of Cry1Ea toxicity and midgut binding sites in *A. gemmatalis* and *C. includens* and sheds light on the cross-resistance potential of Cry1Ea with other Bt proteins aimed at controlling lepidopteran pests in soybeans.

## Introduction

Soybean (Glycine max (L.)), is an important crop that has been increasingly planted worldwide, and reached an annual production of about 363 million tons in 2018–19^1^. Soybean is used directly for feeding or processed to produce soybean meal (for animal feed), oil, and more recently, biodiesel. It was the fourth leading crop (by volume) produced globally in 2016, and the world trade was projected to increase by 22, 20 and 30 percent in soybeans, soybean meal and soybean oil respectively, according to USDA Agricultural Projections to 2025^2^. Soybean is cultivated in diverse climatic zones, from temperate to subtropical and tropical regions. In 2017–18, the United States and Brazil were the major soybean producers (each with about a third of the total world production) followed by Argentina (15%), China (4.4%), India (3.2%), Paraguay (2.5%), and Canada (2%)^1^. Despite the diverse climate conditions of the soya producing areas, soybeans in North and South America are attacked by the lepidopterans *Anticarsia gemmatalis* Hübner (velvetbean caterpillar) (Lepidoptera: Noctuidae) and *Chrysodeixis includens* Walker (soybean looper) (Lepidoptera: Noctuidae), two of the most damaging defoliating caterpillars of soybean^3–5^.

Traditionally, *A. gemmatalis* and *C. includens* have been controlled with chemical insecticides. More recently, the use of alternative insecticides such as biopesticides based on *Bacillus thuringiensis* (Bt) has assumed a prominent position to control insect pests because they have a high degree of specificity, are environmentally friendly, reduce grower costs and reduce the exposure of farmers to hazardous chemicals^6–8^. Advancements in agricultural biotechnology have led to the development of transgenic soybeans expressing the Bt insecticidal protein Cry1Ab in 1994^9^. Since then, other soybean Bt transformed events have been generated^10,11^ and Bt soybean expressing Cry1Ac is currently grown in many countries^12^.

Extensive use of Bt technology without adequate resistance management plans can compromise its durability because the Bt toxin is expressed throughout the plant during the crop cycle, resulting in a high selection pressure that can drive to resistance outbreaks^13^. A way to delay field-evolved resistance is to use Bt crops expressing at least two Bt proteins toxic for the same targets, provided that they do not completely share the same mode of action, a strategy known as pyramiding^14,15^, a key component of an effective resistance management strategy.

Bt proteins kill lepidopteran pests following their specific interaction with receptors found in the insect midgut, leading to pore formation in the apical membrane of the cells, provoking osmotic imbalance and disrupting the gut barrier. Extensive damage, and sometimes a bacterial septicaemia in the hemocoel, result in larval death^16^. The chain of events underlying the toxicity is not fully understood^17–19^ but it is well established that the binding of the Cry toxins to the receptors located in the insect midgut determines the specificity of the Cry toxins and is an essential step for toxicity^20–23^. So far, different proteins have been identified as receptors for Bt in Lepidoptera, including aminopeptidases, alkaline phosphatases, cadherins, glycolipids and ABC transporters^16^.

There has been limited research on the Cry1E Bt protein to date. Its insecticidal activity has been reported for several lepidopterans^24–26^ including soya pests^27^, and studies with Cry1Ac resistant strains suggested no common receptors with Cry1Ac, since a low or no cross-resistance to Cry1E was observed^26,28^. The identity of the Cry1E receptors in the insect midgut remains unknown.

With the aim of finding new insecticidal protein candidates valid for combining as resistance management pyramids in transgenic soya crops, this study investigated the potential of Cry1Ea to control *A. gemmatalis* and *C. includens*. Importantly, we investigated the potential of Cry1Ea to share midgut binding sites with proteins expressed currently in some commercialized transgenic crops, such as Cry1Ac and Cry1F^11^.

## Methods

### Insects and toxicity assays

*A. gemmatalis* and *C. includens* colonies were obtained and reared in the laboratory as described in Bel *et al*.^29^.

Insect bioassays were performed using the diet overlay method^30^. The susceptibility to Cry1Ea protoxin was tested with neonate larvae (24–48 hr old). The *C. includens* and *A. gemmatalis* eggs were obtained as reported in Bel *et al*.^29^. Seven different concentrations of each protein, ranging from 1 to 9,000 ng/cm^2^, and a negative control buffer (10 mM CAPS, pH10) were used in each bioassay experiment. Exposure time was five days. The treatments were carried out using 16 larvae per sample and replicated two times.

In all insect bioassays, the total number of insects exposed to each protein sample, the number of dead insects, and the weight of surviving insects were recorded. Insects that were alive, but that have not grown during the course of the assay and did not respond to perturbation were classified as moribund insects. Dead insects in controls did not exceed 20%.

To estimate the 50% lethal concentration (LC_50_) and 90% lethal concentration (LC_90_) of the dead insects (mortality) or of the dead plus moribund insects (practical mortality), Probit analyses^31^ were conducted using POLO-PC (LeOra Software). Practical mortality was calculated since it displays the full toxicity caused by the protein unlike the mortality, which only takes into account the number of dead insects^32^.

### Cry proteins production and purification

Cry proteins (Cry1Ea, Cry1Ac and Cry1Fa) were expressed in recombinant *Pseudomonas fluorescens* strains as described in Squires *et al*.^33^. Inclusion bodies (IB) were prepared from *Pseudomonas* cell pellets in the following manner. Cells were resuspended to 10% w/v in lysis buffer (50 mM Tris pH 7.5, 200 mM NaCl, 20 mM EDTA disodium salt (Ethylenediaminetetraacetic acid), 0.5% Triton X-100, and 1 mM Dithiothreitol (DTT); 2 mM benzamidine hydrochloride (Sigma-Aldrich B6506)). Cells were gently suspended using a stir plate at room temperature for 10 min. Lysozyme (0.2 mg/ml; Sigma-Aldrich L7651) was added to the cell suspension by mixing with a metal spatula, and the suspension was warmed to 30 °C for 10 min. DNase (DN25; Sigma-Aldrich) was then added at 0.1 mg/ml and MgCl_2_ added to 60 mM to activate the enzyme. Cells were incubated for an additional 15 min at 30 °C. The suspension was cooled on ice for 15 min, then sonicated using a Branson Sonifier 250 (two 1- min sessions, at 70% duty cycle, 30% output). The lysate was centrifuged at 14,000 x g for 40 minutes (4 °C) to pellet IBs. The IB pellet was suspended in 100 ml lysis buffer and homogenized as above. The IB pellet was then repeatedly washed by suspension in 50 ml lysis buffer.

Inclusion bodies were solubilized in 20 mM CAPS (3-(Cyclohexylamino)-1-propanesulfonic acid; Sigma-Aldrich C2632) pH 10 buffer. The solution was centrifuged at 30,000 × g for 30 min at 4 °C, and the resulting supernatant was aliquotted and stored at −80 °C. Cry proteins were trypsinized to generate a Cry protein active “core”. Briefly, bovine trypsin (Sigma-Aldrich; T1426) was added at 1:20 trypsin:protein ratio (w:w), and incubated at 30 °C for 2 hours. Cry protein was further purified by anion exchange chromatography using CAPS pH 10.0 binding buffer and elution with the same buffer containing 1 M NaCl. Cry protein cores typically eluted with 0.3 M NaCl. Purified protein was buffer exchanged to 50 mM CAPS pH 10.0.

### Midgut isolation and Brush Border Membrane Vesicles (BBMV) preparation

*C. includens* and *A. gemmatalis* midguts were dissected from last-instar larvae, washed and frozen in liquid nitrogen as described previously^29^, and preserved at −80 °C until required.

Brush border membrane vesicles (BBMV) were prepared by the differential magnesium precipitation method^34^. The recovered BBMV were suspended in 125 mM mannitol, 8.5 mM Tris, and 2.5 mM EGTA, pH 7.5, aliquoted, snap frozen in liquid nitrogen and stored at −80 °C until used. BBMV protein content was determined by the Bradford assay^35^ using bovine serum albumin (BSA) as a standard.

### Iodination of cry proteins

Labelling of purified truncated proteins was performed with Na^125^I (PerkinElmer Inc., Billerica, MA), using the chloramine T method^36^. The Cry1Ea protein (25 µg) was labelled with either 0.5 mCi or 1 mCi of Na ^125^I following the methodology described by Hernández-Rodríguez *et al*., 2008. Four different labelling reactions were performed. The estimated specific activities of the labelled protein in each labelling assay was obtained based on the input toxin concentration, the radioactivity eluted in the protein peak and the percentage of radioactivity observed in the Cry1Ea band with respect to the radioactivity in other minor bands revealed after SDS-PAGE^37^. The estimated specific activities obtained in the present work ranged from 0.1 to 5.1 µCi/µg Cry1Ea. The differences in specific activities did not affect the binding results.

### Binding assays

The binding assays were performed as described in Bel *et al*.^29^. The BBMV optimum concentration to be used in the competition binding experiments was determined by incubation of 0.3 nM of the labelled toxin, with increasing amounts of BBMV (ranging from 0.05 to 0.3 mg/ml), for 1 h, at room temperature. An excess of unlabelled toxin (0.3 µM) was used to determine the non-specific binding; the concentration of the unlabelled protein used accounts for 1000-fold the concentration of the labelled protein, an excess for which we assume that all receptors will be occupied^38,39^. Radioactivity associated to the BBMV was measured in a Gamma counter (2480 WIZARD2 Automatic Gamma Counter, PerkinElmer, Downers Grove, IL, USA). Each experiment was repeated at least two times. The optimal concentration of BBMV in the binding assays was set at 0.2 mg BBMV/ml for both insect species (determined as a compromise between an acceptable specific binding signal and the necessity of maintaining the reaction in the linear range). To determine the optimum reaction time for binding experiments, time course experiments were performed with BBMV of both insect species; after the analyses, reaction times were set at 60 min for both insect assays, time in which the respective reactions were in the steady-state or close enough to it to have satisfactory values of specific binding.

Homologous and heterologous competition experiments were performed by adding increasing concentrations of unlabelled proteins to the binding reaction tubes that contained the labelled Cry1E and 0.2 mg/ml of *A. gemmatalis* or *C. includens* BBMV in a final volume of 0.1 ml. Each competition experiment was repeated at least twice. The binding parameters *K*_*d*_ (dissociation constant, inversely correlated with affinity) and *R*_*t*_ (concentration of binding sites) were obtained using the LIGAND software^40^. Graphic representations of the competition curves were performed with the GraphPad Prism Version 5.00 for Windows (GraphPad Software, San Diego, CA).

## Results

### Susceptibility of *A. gemmatalis* and *C. includens* to Cry1Ea

Results from bioassays conducted with *A. gemmatalis* and *C. includens* to assess the toxicity of Cry1Ea are summarized in Table 1. *C. includens* was more susceptible to this toxin in terms of mortality as the LC_50_ was calculated as 46 ng/cm^2^, whereas the LC_50_ versus *A. gemmatalis* was calculated as 1311 ng/cm^2^. However, the LC_50_ value for practical mortality, which accounts for moribund insects, suggests that *C. includens* (LC_50 = _14 ng/cm^2^) and *A. gemmatalis* (LC_50_ = 22 ng/cm^2^) survivability to Cry1Ea is nearly identical.

**Table 1:** Toxicity of Cry1Ea to neonate larvae of *A. gemmatalis* and *C. includens*. Parameters were obtained by Probit analyses based on the number of dead larvae (mortality) and the number of dead and moribund larvae (practical mortality).

Species	Mortality				Practical mortality (dead & moribund)			
	LC_50_	FL_95%_	LC_90_	FL_95%_	LC_50_	FL_95%_	LC_90_	FL_95%_
*C. includens*	46	35–59	216	154–346	14	8–20	83	57–146
*A. gemmatalis*	1311	740–2731	>9000	NA	22	16–29	63	46–109

Data in the table are expressed as ng/cm^2^. FL_95%_, Fiducial limits at the 95% level.^1^LC_50_, 50% lethal concentration;^2^LC_90_, 90% lethal concentration.

### Specific binding of Cry1Ea to *A. gemmatalis* and *C. includens* BBMV

**Figure 1:**
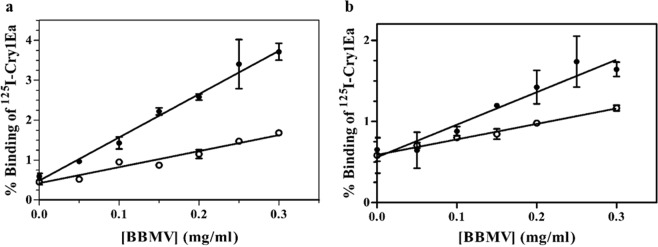
Binding of Cry1Ea at increasing concentrations of BBMV proteins. (**a**) *A. gemmatalis* (**b**) *C. includens*. ●, Total binding; ○, non-specific binding. Results represent the mean and standard deviation of two or three replicates with several duplicated points.

The binding of Cry1Ea to *A. gemmatalis* and *C. includens* BBMV was determined by incubating a fixed concentration of ^125^I-Cry1Ea (3 nM) with increasing concentrations of the corresponding insect BBMV. Specific binding was obtained in both insect species and was calculated by subtracting the non-specific binding (determined by adding an excess of unlabeled Cry1Ea to the binding reactions) from the total binding (Fig. 1). Results showed that *A. gemmatalis* BBMV had higher specific binding values than *C*.

### Competitive binding of ^125^I-Cry1Ea to *A. gemmatalis* and *C. includens* BBMV

**Figure 2:**
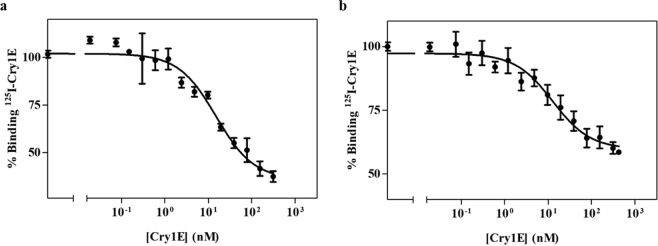
Homologous competition binding experiments with ^125^I-Cry1Ea. Curves represent total binding of ^125^I-Cry1Ea at increasing concentrations of unlabeled competitor, using BBMV from *A. gemmatalis* (**a**) or from *C. includens* (**b**). Data points represent the mean of two to five replicates performed with four different batches of labelled protein.

**Figure 3:**
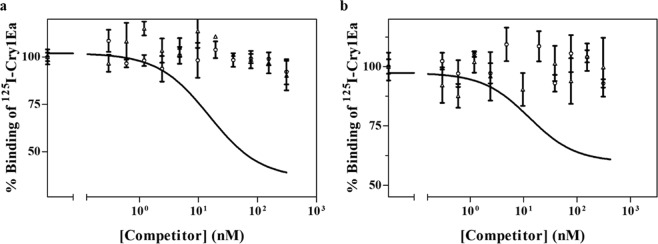
Heterologous competition binding experiments with ^125^I-Cry1Ea. Curves represent total binding of ^125^I-Cry1Ea at increasing concentrations of unlabeled competitor, using BBMV from *A. gemmatalis* (**a**) or from *C. includens* (**b**). Data points represent the mean of two independent replicates. Open circles: Cry1Ac; open triangles: Cry1Fa.

Competition experiments were performed by incubating the insect BBMV with ^125^I-Cry1Ea in the presence of increasing amounts of non-labelled Cry1Ea (for homologous competition experiments) or Cry1Ac and Cry1Fa (for heterologous competition assays). The BBMV concentration selected to perform the competition binding experiments was 0.2 mg/ml for both insect species (see Materials and methods section). The homologous and heterologous competition binding results for both insects are summarized in Figs. 2 and 3 respectively.

Homologous competition data obtained for both insects was consistent with the occurrence of a single population of Cry1Ea binding sites (Fig. 2). A high level of nonspecific binding was observed: it accounted for more than 55% of total binding in *C. includens* BBMV and for about 38% of total binding in *A. gemmatalis* BBMV. Dissociation constants (*K*_*d*_) and concentration of binding sites (*R*_*t*_) were estimated from the homologous competition results and are summarized in Table 2.

**Table 2:** Cry1Ea binding parameters (*K*_*d*_ and *R*_*t*_) for *A. gemmatalis* and *C. includens*. Results represent the Mean ± SEM of 3 to 4 replicates, using four independent ^125^I-Cry1Ea batches.

	*K*_*d*_ (nM)	*R*_*t*_ (pmol/mg)^a^
*A. gemmatalis*	17.6 ± 2.8	2.5 ± 0.3
*C. includens*	21.2 ± 3.4	0.8 ± 0.1

^a^Values are expressed in picomoles per milligram of BBMV protein.

The results of heterologous binding experiments (Fig. 3) showed that Cry1Ac and Cry1Fa did not compete for the Cry1Ea binding sites, in either *A. gemmatalis* or C*. includens*. The reciprocal experiments, performed with labelled Cry1Ac and Cry1Fa (Supplementary Material Fig. 1), displayed the expected complementary results, showing no competition of Cry1Ea for Cry1Ac or Cry1Fa binding sites.

## Discussion

*A. gemmatalis* and *C. includens* are two important soybean pests that at present are controlled with transgenic soybean varieties that express the *B. thuringiensis* insecticidal protein Cry1Ac^5,12,41,42^. Also, other soybean events expressing the Bt proteins Cry1Ac, Cry1F, Cry2A and the chimera Cry1A105 (that combines the domains I and II of Cry1Ab or Cry1Ac, and domain III of Cry1F) either alone or pyramided, have been approved for cultivation in many countries^11^. The main threat to the sustainability of Bt technology is the development of resistance, which can be hastened by high selection pressure of extensive soya monocultures. This scenario could be delayed, for example, by pyramiding multiple insecticidal Bt proteins that do not share binding receptors in the insect midguts^14,15^.

In the current study, we have evaluated Cry1Ea as an additional means to control *A. gemmatalis* and *C. includens*. As presented in Table 1, Cry1Ea has potent activity against both insect species in terms of practical mortality, which accounts for dead insects as well as moribund insects that have not developed beyond the first instar. However, in terms of lethal activity, *C. includens* was more susceptible to Cry1Ea as the LC_50_ was 46 ng/cm^2^ compared to 1311 ng/cm^2^ measured for *A. gemmatalis*. While we do not understand the underlying cause of this discrepancy, practical mortality (sometimes referred to as functional mortality) is frequently employed as measure to understand the efficacy of an insecticidal protein to provide plant protection in the field. Thus, these data indicate that both *A. gemmatalis* and *C. includens* are both sensitive to Cry1Ea at agronomically feasible levels. The level of *in vitro* susceptibility to Cry1Ea is similar to other Cry1 Bt proteins that have been successfully introduced into plants to provide protection from Lepidopteran pests^27,29,43,44^. Therefore, Cry1Ea has insecticidal potential to provide protection against these two soybean pests.

Next, we evaluated the potential of cross-resistance to other Cry genes currently expressed in soybean to determine the suitability of Cry1Ea in gene pyramiding strategies. In this work, we utilized binding studies and inferred membrane binding site models add prognostic value towards the possibility of cross-resistance. The utility of binding studies for cross-resistance evaluation, in *C. includens*, has recently been evidenced in an study by Rodrigues-Silva *et al*.^44^ with a strain resistant to Cry1Ac selected in the laboratory, in which they observed absence of cross-resistance to Cry1Fa or Cry2Aa, which had been previously predicted by binding studies^29^.

Binding results with ^125^Iodine-labelled Cry1Ea showed that this protein bound specifically to both, *A. gemmatalis* and *C. includens* BBMV. The homologous competition binding results allowed the calculation of the binding parameters and revealed that *K*_*d*_ values are higher (from 2 to 260 fold) than the ones obtained for Cry1Ac and Cry1Fa proteins in these insect species^29^, and in general for Cry1A, Cry1F and Cry2 proteins in lepidopteran pests^37,45–47^, which indicate that Cry1Ea binds to *A. gemmatalis* or to *C. includens* BBMV receptors with lower affinity than Cry1Ac or Cry1Fa. On the other hand, *R*_*t*_ values are in the range of values described for Cry1Ac and Cry1Fa^29^, indicating that Cry1Ea has a number of binding sites in the range of the ones found for Cry1Ac and Cry1Fa in these insect pests.

The heterologous competition binding results showed that in both *A. gemmatalis* and in *C. includens*, Cry1Ac and Cry1Fa did not compete for Cry1Ea binding sites (Fig. 3), and reciprocally, Cry1Ea showed lack of binding competition for Cry1Ac or Cry1Fa binding sites (Suppl. Fig. 1). These data show the absence of common binding sites amongst Cry1Ea and Cry1Ac or Cry1Fa. Differential binding sites of Cry1A and Cry1E have already been observed in some lepidopteran species (*Heliothis virescens, Manduca sexta, S. littoralis* and *S. frugiperda*)^21,48,49^. Thus, our data are consistent with extant literature suggesting a unique mode of action for Cry1Ea.

In summary, the results presented in this paper indicate that pyramiding Cry1Ea in transgenic soybean crops could be a suitable option to delay insect resistance outbreaks. The binding model obtained from the *in vitro* binding assays, will be completed in future studies focused on identifying and understanding the nature of the Cry1 binding sites in the receptors. This information will help predict the durability of the Cry protein pyramids to control these two soybean pests.

## Supplementary information

Figure S1
