# Supplementary material for: Specific binding of Bacillus thuringiensis Cry1Ea toxin, and Cry1Ac and Cry1Fa competition analyses in Anticarsia gemmatalis and Chrysodeixis includens
Source: Sci Rep. 2019 Dec 3;9:18201. doi: 10.1038/s41598-019-54850-3 (PMC6890801; doi:10.1038/s41598-019-54850-3)
Supplement: Supplementary file 1 — Figure S1 [file 41598_2019_54850_MOESM1_ESM.pdf]

Supplementary Figures

Specific binding of *Bacillus thuringiensis* Cry1Ea toxin and Cry1Ac and Cry1Fa competition analyses in *Anticarsia gemmatalis* and *Chrysodeixis includens*.

Yolanda Bel[a,], Marc Zack[b] , Ken Narva[b], Baltasar Escriche[a,]

[a]ERI de Biotecnología y Biomedicina (BIOTECMED), Department of Genetics, Universitat de València, 46100 Burjassot, Spain

[b]Dow AgroSciences, Indianapolis, Indiana, USA

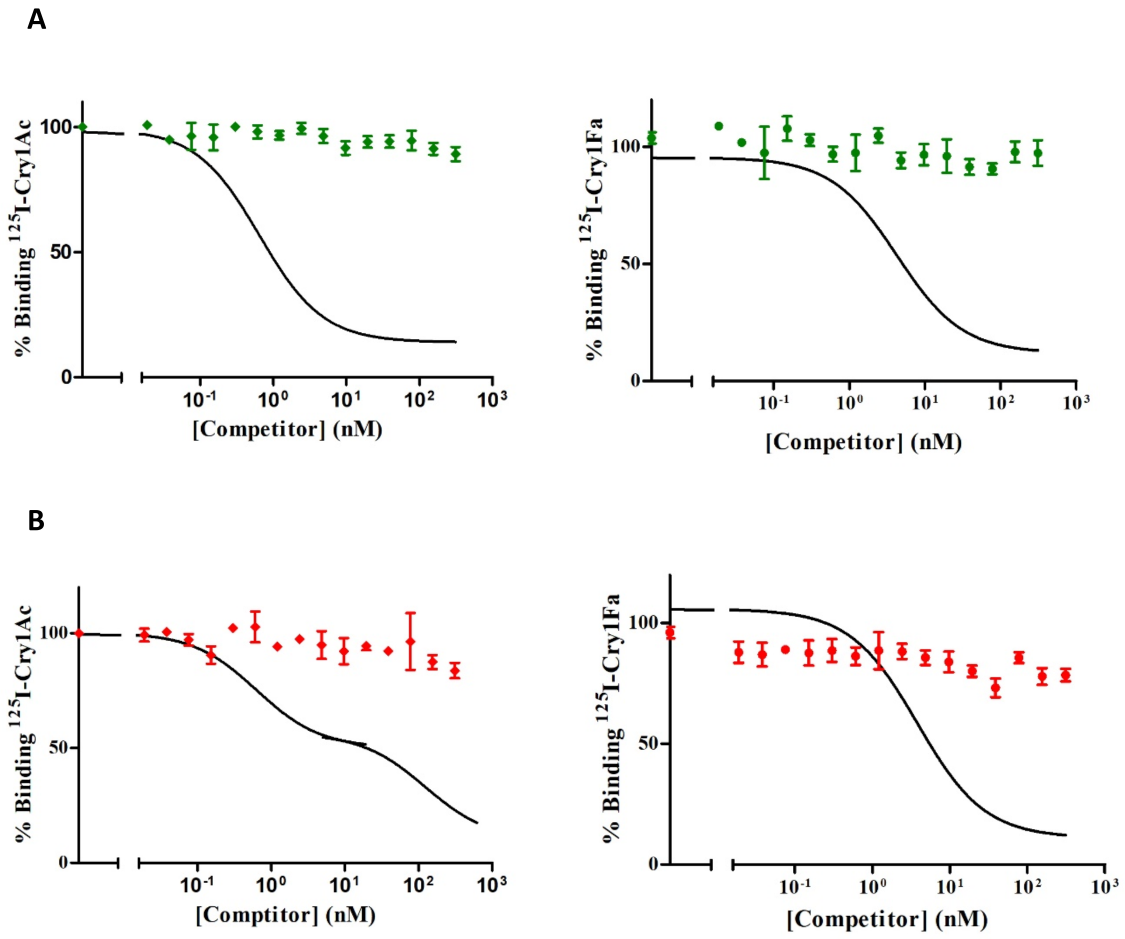

Figure S1. Competitive binding of Cry1Ea for ^{125}I-Cry1Ac or ^{125}I-Cry1Fa binding sites in *A. gemmatalis* (A) and *C. includens* (B). Cry1Ac and Cry1Fa labeling and biding experiments were performed as described previously[29], using about 3 nM of ^{125}I-Cry1Ac or ^{125}I-Cry1Fa, and incubating with the corresponding insect BBMV at room temperature, for 90 min and 50 min respectively.The results represent the mean of duplicated experiments performed with two different batches of labeled proteins.
